# Cancer mortality predictions for 2025 in Latin America with focus on prostate cancer

**DOI:** 10.1097/CEJ.0000000000000959

**Published:** 2025-02-25

**Authors:** Silvia Mignozzi, Claudia Santucci, Fabio Levi, Matteo Malvezzi, Paolo Boffetta, Giovanni Corso, Eva Negri, Carlo La Vecchia

**Affiliations:** a Department of Clinical Sciences and Community Health, University of Milan, Milan, Italy; b Department of Epidemiology and Health Services Research, Centre for Primary Care and Public Health (Unisanté), University of Lausanne, Lausanne, Switzerland; c Department of Medicine and Surgery, University of Parma, Parma, Italy; d Department of Family, Population & Preventive Medicine, Stony Brook University, Stony Brook, New York, USA; e Department of Medical and Surgical Sciences, University of Bologna, Bologna; f Division of Breast Surgery, European Institute of Oncology (IEO), Istituto di Ricovero e Cura a Carattere Scientifico (IRCCS); g Department of Oncology and Hemato-Oncology, University of Milan, Milan, Italy

**Keywords:** cancer mortality, Latin America, predictions, prostate cancer, screening, tobacco

## Abstract

We provided cancer mortality rate estimates for the year 2025 in six Latin American countries (Argentina, Brazil, Chile, Colombia, Cuba, and Mexico), focusing on prostate cancer. We extracted mortality data for all cancers combined and the most common sites from the WHO and population data since 1970 from the United Nations. Estimates for 2025 were computed applying a linear regression to the most recent segment identified through Poisson join-point regression. Avoided deaths number from 1991 to 2025 was estimated by applying the 1990 peak rate to population data. Mortality from all cancers is predicted to be favorable for both sexes in all countries. The lowest total cancer mortality rates are expected in Mexico (67.7/100 000 males; 61.4/100 000 females), while the highest ones in Cuba (136.6/100 000 males; 91.6/100 000 females). Prostate cancer mortality is declining in all countries, although rates remain high in Cuba (25.2/100 000 in 2025). Downward patterns are observed for all age groups in all countries, except the elderly in Cuba and Mexico. Declines in mortality are predicted for colorectal (except for males in Brazil and Cuba, and females in Chile), stomach (except Cuban males), pancreatic (except Argentinian and Cuban males), lung, bladder (except Argentinian females), breast, and ovarian (except Cuba) cancers. Uterine cancer mortality, particularly from cervical cancer, remains highin Argentina (10.2/100 000) and Cuba (10.4/100 000). Except for uterine, stomach, and prostate cancers, cancer mortality rates are still relatively low in Latin America, except Cuba. Controlling tobacco particularly in Cuba, implementing organized cervical cancer screening, and advancing cancer treatment also for prostate cancer remain crucial in all countries considered.

## Introduction

Since 2017, we have provided cancer mortality predictions for selected major Latin American countries with good quality data (Carioli *et al*., 2017, 2020, 2022; Santucci *et al*., 2023). Past projected figures showed declines in overall cancer mortality in Argentina, Brazil, Chile, Colombia, and Mexico but less in Cuba and Venezuela. Stomach and cervical cancer still maintain comparatively high rates, while colorectal cancer mortality is comparatively low, except in Argentina and Cuba.

Cancer mortality estimates help to quantify the burden of cancer and also to monitor the impact of incidence, stage at diagnosis, and treatment on mortality. In the present work, we therefore present predicted numbers and rates of cancer deaths for the year 2025, with detailed analyses and discussions on prostate cancer.

## Materials and methods

We retrieved official death certification data for the following cancer sites: stomach, colorectum, pancreas, lung, breast, uterus (including cancer of the cervix, endometrium, and unspecified site in the uterus), ovary, prostate, bladder, and leukemias, and all cancers combined from the WHO database (World Health Organization mortality database, 2024). Corresponding International Classification of Diseases, 10th Revision codes are provided in Supplementary Table 1, Supplemental digital content 1, http://links.lww.com/EJCP/A546. Population estimates were obtained from the United Nations database (United Nations, 2024). We considered Argentina, Brazil, Chile, Colombia, Cuba, and Mexico. Those countries have good data coverage (≥90%) and high data quality (medium for Argentina), as defined by the WHO (WHO, 2020). However, we were unable to update our estimates for Venezuela, since the latest available death data were for 2016.

For each cancer site and country considered, we derived sex-specific and 5-year age-specific mortality rates from 1970 to the most recent year available. We obtained age-standardized mortality rates (ASRs) using the world standard population and analyzed ASR trends by fitting joinpoint regression models (Kim *et al*., 2000, 2022).

To obtain projected ASRs for 2025, we first fitted a log Poisson joinpoint regression model to the observed number of deaths in each 5-year age group for each cancer site. We then estimated the age-specific number of deaths for 2025, with corresponding 95% prediction intervals, by fitting a linear regression model to the mortality figures for each age group over the most recent trend segment identified by the join-point model. We then calculated age-specific and age-standardized death rates (and associated 95% prediction intervals) using the estimated age-specific number of deaths and the projected population from the UN database (United Nations, 2024) for the year 2025. We also estimated the number of deaths averted for each country for the period 1991–2025 by comparing observed and predicted deaths with those expected based on the 1990 age-specific peak rate.

Ethics committee approval was not required as only anonymous public data were used.

All analyses were performed using the software R version 4.3.2 (R Core Team 2023, Vienna, Austria), SAS version 9.4 (SAS Institute Inc., Cary, North Carolina, USA), and Joinpoint Regression Program version 5.3.0.0 - November 2024 (Statistical Methodology and Applications Branch, Surveillance Research Program, National Cancer Institute, Bethesda, Maryland, USA).

## Results

**Fig. 1:**
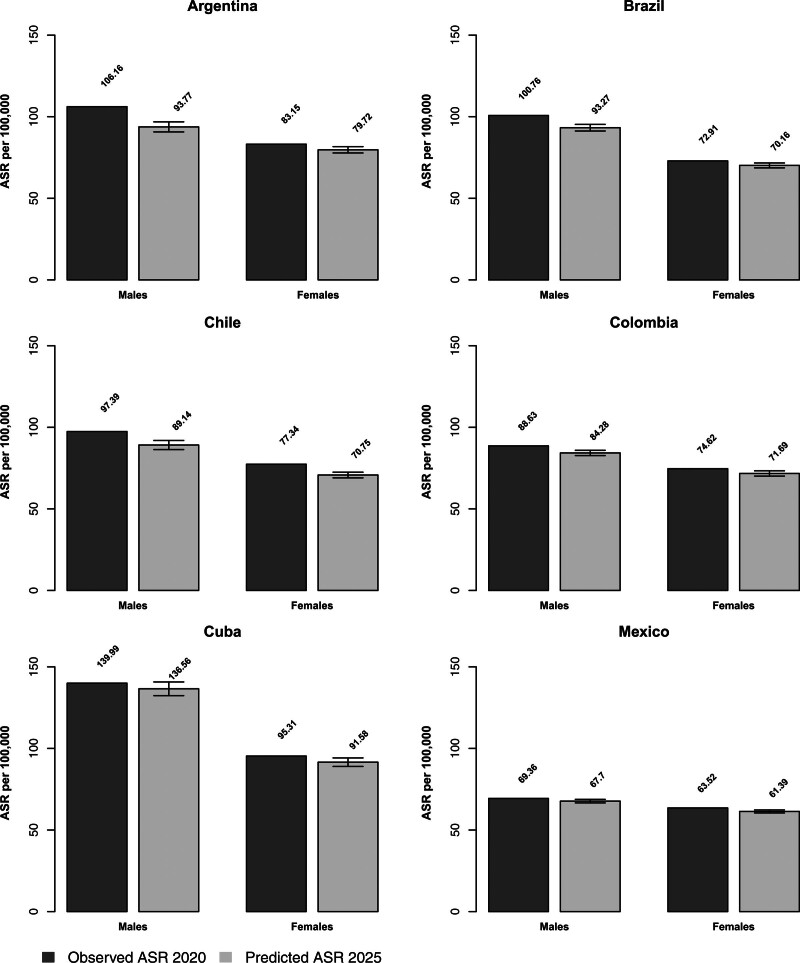
Bar plots of age-standardized (world population) mortality rates (ASR) per 100 000 persons for the year 2020 and predicted ASR for 2025 with 95% prediction intervals for all cancers combined according to sex in Argentina, Brazil, Chile, Colombia, Cuba, and Mexico.

Table 1 and Table 2 give the number of predicted cancer deaths and ASRs per 100 000 with the corresponding 95% prediction intervals in the six selected Latin American countries for the year 2025, compared with the observed data in 2020, for males and females, respectively. Figure 1 shows bar plots of ASRs per 100 000 in 2020 and predicted ASRs for 2025 with corresponding 95% prediction intervals from all cancers combined for each country considered, according to sex.

**Table 1:** Number of predicted deaths and mortality rates (ASRs) per 100 000 males for the year 2025 and comparison figures for 2020, from the six selected Latin American countries, with 95% prediction intervals (PIs) and percent differences between ASRs

Cancer	Observed number of deaths in 2020	Predicted number of deaths in 2025 (95% PI)	Observed ASR in 2020	Predicted ASR in 2025 (95% PI)	% Difference 2025 vs 2020
Argentina					
Stomach	1664	1710 (1605–1815)	5.74	5.46 (5.12–5.80)	−4.88
Colorectum	4104	3990 (3836–4153)	13.93	12.53 (12.02–13.03)	−10.05
Pancreas	2072	2280 (2173–2382)	7.12	7.21 (6.87–7.55)	1.26
Lung	5542	4490 (4184–4792)	19.10	13.75 (12.69–14.80)	−28.01
Prostate	3513	3460 (3264–3651)	10.54	9.50 (8.96–10.03)	−9.87
Bladder	992	1020 (950–1093)	3.12	2.97 (2.75–3.19)	−4.81
Leukemias	973	1000 (927–1078)	3.56	3.45 (3.16–3.73)	−3.09
All cancers	31 100	29 890 (28 968–30 813)	106.16	93.77 (90.68–96.85)	−11.67
Brazil					
Stomach	8772	8780 (8400–9152)	7.24	6.24 (5.97–6.51)	−13.81
Colorectum	12 726	15 270 (14 813–15 720)	10.65	10.89 (10.54–11.24)	2.25
Pancreas	5880	6410 (6147–6668)	4.87	4.54 (4.35–4.73)	−6.78
Lung	16 007	16 310 (15 852–16 766)	13.30	11.44 (11.09–11.80)	−13.98
Prostate	15 841	17 410 (17 086–17 741)	14.00	12.64 (12.41–12.87)	−9.71
Bladder	3097	3540 (3396–3689)	2.71	2.58 (2.46–2.70)	−4.80
Leukemias	3705	3870 (3674–4072)	3.29	3.09 (2.94–3.25)	−6.08
All cancers	119 209	129 090 (126 356–131 818)	100.76	93.27 (91.25–95.28)	−7.43
Chile					
Stomach	2132	1930 (1811–2046)	14.08	11.02 (10.32–11.73)	−21.73
Colorectum	1700	1750 (1641–1860)	11.20	10.05 (9.43–10.67)	−10.27
Pancreas	720	800 (741–851)	4.87	4.63 (4.30–4.95)	−4.93
Lung	1982	2150 (2042–2264)	13.06	11.97 (11.31–12.63)	−8.35
Prostate	2104	2400 (2262–2531)	11.88	11.50 (10.90–12.10)	−3.20
Bladder	388	430 (387–467)	2.38	2.27 (2.05–2.49)	−4.62
Leukemias	390	400 (365–443)	3.06	2.88 (2.51–3.24)	−5.88
All cancers	14 898	15 890 (15 360–16 409)	97.39	89.14 (86.34–91.94)	−8.47
Colombia					
Stomach	3198	3520 (3384–3652)	11.54	10.84 (10.42–11.25)	−6.07
Colorectum	2245	2330 (2185–2473)	8.16	7.08 (6.59–7.56)	−13.24
Pancreas	1041	1130 (1060–1199)	3.75	3.47 (3.26–3.69)	−7.47
Lung	2730	2650 (2495–2813)	9.92	8.13 (7.66–8.59)	−18.04
Prostate	3493	3910 (3769–4048)	13.07	12.17 (11.71–12.63)	−6.89
Bladder	409	420 (377–457)	1.52	1.28 (1.15–1.41)	−15.79
Leukemias	1011	1060 (992–1124)	3.88	3.68 (3.43–3.94)	−5.15
All cancers	24 163	26 930 (26 412–27 437)	88.63	84.28 (82.67–85.89)	−4.91
Cuba					
Stomach	539	570 (527–622)	4.84	4.98 (4.53–5.44)	2.89
Colorectum	1340	1480 (1405–1560)	11.77	12.06 (11.39–12.73)	2.46
Pancreas	485	540 (498–587)	4.50	4.78 (4.35–5.22)	6.22
Lung	3451	3140 (2936–3345)	31.47	25.45 (23.56–27.35)	−19.13
Prostate	3484	3690 (3491–3886)	26.04	25.24 (24.00–26.49)	−3.07
Bladder	537	550 (498–608)	4.40	4.26 (3.80–4.72)	−3.18
Leukemias	292	300 (261–342)	3.49	3.34 (2.72–3.95)	−4.30
All cancers	15 630	16 640 (16 141–17 128)	139.99	136.56 (132.37–140.75)	−2.45
Mexico					
Stomach	3435	3560 (3429–3691)	5.10	4.71 (4.54–4.88)	−7.65
Colorectum	4353	4500 (4280–4711)	6.54	5.97 (5.67–6.27)	−8.72
Pancreas	2307	2580 (2470–2687)	3.49	3.44 (3.29–3.59)	−1.43
Lung	4056	3820 (3635–4014)	5.97	4.91 (4.66–5.17)	−17.76
Prostate	7896	8100 (7824–8369)	10.60	9.72 (9.4–10.03)	−8.30
Bladder	851	860 (798–917)	1.20	1.07 (1.00–1.15)	−10.83
Leukemias	2454	2660 (2547–2774)	3.79	3.89 (3.71–4.07)	2.64
All cancers	47 013	51 270 (50 476–52 062)	69.36	67.70 (66.64–68.76)	−2.39

**Table 2:** Number of predicted deaths and mortality rates (ASRs) per 100 000 females for the year 2025 and comparison figures for 2020, from the six selected Latin American countries, with 95% prediction intervals (PI) and percent differences between ASRs

Cancer	Observed number of deaths in 2020	Predicted number of deaths in 2025 (95% PI)	Observed ASR in 2020	Predicted ASR in 2025 (95% PI)	% Difference 2025 vs 2020
Argentina					
Stomach	985	990 (912–1067)	2.73	2.56 (2.37–2.75)	−6.23
Colorectum	3573	3600 (3455–3738)	8.97	8.79 (8.46–9.12)	−2.01
Pancreas	2148	2290 (2128–2454)	5.35	5.35 (5.06–5.65)	0.00
Lung	3075	3190 (3038–3347)	8.56	8.13 (7.70–8.55)	−5.02
Breast	5563	5840 (5547–6132)	15.95	15.89 (15.26–16.52)	−0.38
Uterus	2920	3280 (3163–3405)	9.56	10.16 (9.76–10.56)	6.28
Ovary	1316	1290 (1204–1366)	4.07	3.64 (3.40–3.88)	−10.57
Bladder	325	340 (298–389)	0.68	0.69 (0.60–0.77)	1.47
Leukemias	720	770 (689–842)	2.31	2.29 (2.07–2.50)	−0.87
All cancers	29 962	30 320 (29 379–31 268)	83.15	79.72 (77.78–81.66)	−4.13
Brazil					
Stomach	5078	5470 (5281–5668)	3.28	3.11 (2.99–3.22)	−5.18
Colorectum	13 205	15 290 (14 932–15 657)	8.48	8.45 (8.26–8.64)	−0.35
Pancreas	6011	6820 (6588–7054)	3.80	3.67 (3.54–3.79)	−3.42
Lung	12 608	13 580 (13 040–14 123)	8.18	7.45 (7.13–7.77)	−8.92
Breast	17 823	19 770 (19 277–20 264)	12.09	11.81 (11.5–12.11)	−2.32
Uterus	10 393	11 700 (11 403–11 994)	7.13	7.19 (7.00–7.37)	0.84
Ovary	3975	4440 (4267–4612)	2.70	2.63 (2.52–2.74)	−2.59
Bladder	1498	1650 (1570–1719)	0.92	0.84 (0.80–0.88)	−8.70
Leukemias	3034	3130 (2964–3294)	2.22	2.04 (1.92–2.16)	−8.11
All cancers	110 077	121 850 (119 261–124 432)	72.91	70.16 (68.66–71.65)	−3.77
Chile					
Stomach	1055	1060 (986–1131)	5.49	4.92 (4.55–5.29)	−10.38
Colorectum	1596	1953 (1853–2054)	8.59	9.09 (8.66–9.51)	5.82
Pancreas	881	960 (906–1013)	4.60	4.47 (4.19–4.75)	−2.83
Lung	1414	1530 (1439–1624)	7.65	7.08 (6.61–7.54)	−7.45
Breast	1644	1700 (1600–1792)	10.02	8.87 (8.26–9.47)	−11.48
Uterus	1108	1140 (1055–1234)	7.09	6.60 (6.01–7.19)	−6.91
Ovary	526	570 (523–617)	3.50	3.33 (3.02–3.64)	−4.86
Bladder	188	210 (184–232)	0.86	0.85 (0.74–0.96)	−1.16
Leukemias	354	380 (344–418)	2.21	2.25 (1.94–2.56)	1.81
All cancers	13 758	14 660 (14 297–15 030)	77.34	70.75 (69.01–72.49)	−8.52
Colombia					
Stomach	2041	2330 (2225–2436)	5.99	5.91 (5.64–6.18)	−1.34
Colorectum	2487	2529 (2383–2675)	7.23	6.21 (5.84–6.58)	−14.11
Pancreas	1154	1280 (1223–1343)	3.30	3.11 (2.96–3.26)	−5.76
Lung	1912	2020 (1889–2158)	5.52	4.88 (4.58–5.18)	−11.59
Breast	3671	3680 (3476–3890)	11.32	9.98 (9.42–10.53)	−11.84
Uterus	2576	2910 (2789–3027)	7.92	7.78 (7.44–8.13)	−1.77
Ovary	1210	1300 (1223–1369)	3.77	3.48 (3.28–3.68)	−7.69
Bladder	182	210 (184–232)	0.50	0.48 (0.42–0.53)	−4.00
Leukemias	822	910 (832–985)	2.73	2.85 (2.57–3.12)	4.40
All cancers	24 944	27 940 (27 313–28 571)	74.62	71.69 (70.07–73.32)	−3.93
Cuba					
Stomach	368	380 (345–423)	2.79	2.70 (2.37–3.02)	−3.23
Colorectum	1668	1730 (1648–1819)	12.07	11.21 (10.60–11.81)	−7.13
Pancreas	460	480 (436–531)	3.52	3.44 (3.11–3.78)	−2.27
Lung	2166	2300 (2186–2405)	17.70	16.26 (15.33–17.20)	−8.14
Breast	1714	1850 (1750–1951)	14.33	13.91 (12.93–14.89)	−2.93
Uterus	1248	1300 (1224–1376)	11.07	10.42 (9.58–11.26)	−5.87
Ovary	369	410 (366–450)	3.48	3.74 (3.27–4.20)	7.47
Bladder	204	200 (176–227)	1.44	1.28 (1.10–1.46)	−11.11
Leukemias	272	240 (212–277)	2.85	2.38 (1.81–2.94)	−16.49
All cancers	11 564	12 310 (12 033–12 594)	95.31	91.58 (88.97–94.20)	−3.91
Mexico					
Stomach	3159	3230 (3119–3348)	4.05	3.61 (3.48–3.75)	−10.86
Colorectum	3615	3780 (3616–3949)	4.62	4.15 (3.95–4.34)	−10.17
Pancreas	2604	2790 (2679–2891)	3.33	3.04 (2.91–3.16)	−8.71
Lung	2683	2800 (2678–2913)	3.38	3.00 (2.86–3.14)	−11.24
Breast	7685	8390 (8180–8602)	10.31	9.88 (9.64–10.13)	−4.17
Uterus	5493	5880 (5700–6061)	7.34	6.98 (6.77–7.19)	−4.90
Ovary	2876	2750 (2552–2952)	3.93	3.27 (3.02–3.51)	−16.79
Bladder	348	350 (304–397)	0.40	0.34 (0.30–0.39)	−15.00
Leukemias	2123	2170 (2058–2280)	3.05	2.92 (2.76–3.08)	−4.26
All cancers	48 448	53 770 (53 016–54 519)	63.52	61.39 (60.47–62.30)	−3.35

Male mortality from all neoplasms combined is favorable in all countries considered, with decreases in ASRs between 2020 and 2025 ranging from −2.4% in Mexico and −2.5% in Cuba to −11.7% in Argentina (Table 1 and Fig. 1). Numbers of deaths for all neoplasms were rising in all countries except for Argentina where they were falling in males and stable in females. Cuba had the highest rates in both periods, with an ASR of 140.0/100 000 in 2020 and a projected 136.6/100 000 in 2025, while Mexico had the lowest ones, with 69.4/100 000 in 2020 and a projected 67.7/100 000 in 2025. In 2025, lung cancer is projected to be the leading cause of cancer death for males in Argentina (13.8/100 000), Chile (12.0/100 000), and Cuba (25.5/100 000), while prostate cancer will be the leading cause in Brazil (12.6/100 000), Colombia (12.2/100 000) and Mexico (9.7/100 000) (Table 1). Colorectal cancer rates varied between 10.0/100 000 and 13.0/100 000 in all countries except Colombia (7.1) and Mexico (6.0). No consistent trend across various countries considered was observed for pancreatic cancer, with rates of around 4.0/100 000 in almost all countries and 7.2 in Argentina, with some upward and downward trends.

Overall female cancer mortality is projected to decrease in all countries considered, with decreases ranging from −3.4% in Mexico to −8.5% in Chile (Table 2 and Fig. 1). Cuba reported the highest ASRs for mortality from all cancers combined: 95.3/100 000 observed in 2020 and 91.6/100 000 projected for 2025, while Mexico had the lowest ones: 63.5/100 000 in 2020 and 61.4/100 000 for 2025. Breast cancer is predicted to be the leading cause of cancer death in 2025 in Argentina (15.9/100 000), Brazil (11.8/100 000), Colombia (10.0/100 000), and Mexico (9.9/100 000), while colorectal cancer is the leading cause of cancer death in Chile (9.1/100 000) and lung cancer in Cuba (16.3/100 000). Uterine cancer, including cervical cancer, is the second leading cause of cancer death in Argentina (10.2/100 000), Colombia (7.8/100 000), and Mexico (7.0/100 000). As for males, colorectal cancer rates were lower in Mexico than in other countries and no consistent trend across various countries considered was observed in pancreatic cancer.

Compared with 2020, we predicted increases in mortality rates from uterine cancer in females in Argentina (+6.3%), ovarian cancer in Cuba (+7.5%), colorectal cancer in Chile (+5.8%), and leukemias in Chile (+1.8%) and Colombia (+4.4%) (Table 2).

**Fig. 2:**
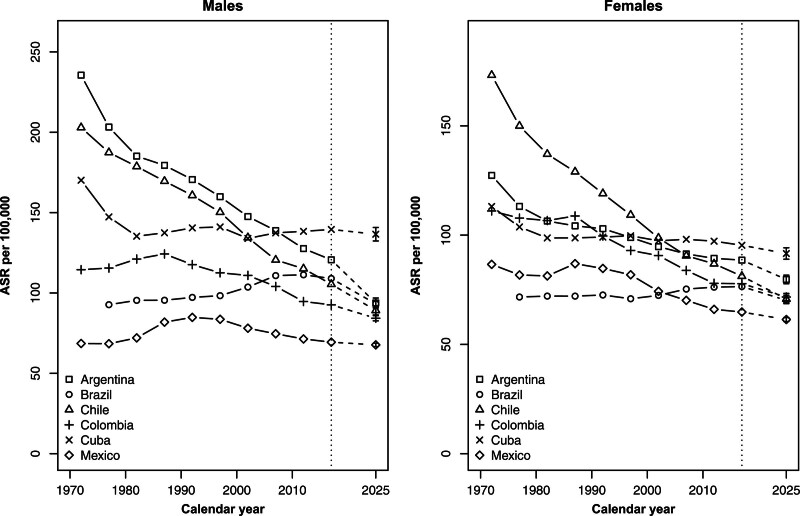
Age-standardized (world population) mortality rates (ASRs) per 100 000 males (left) and females (right) in quinquennia from 1970 to 2020 and predicted ASRs for 2025, with 95% prediction intervals for all cancers combined in Argentina, Brazil, Chile, Colombia, Cuba, and Mexico.

Figure 2 shows the trends in total cancer mortality in the 5-year periods by sex from 1970–1974 to 2015–2019, and the projected rates for 2025 with the corresponding prediction intervals. There was a tendency toward decreasing differences in overall cancer mortality across countries, particularly among females. For males, rates decreased in Argentina and Chile over the whole period. Trends for Colombia and Mexico started to decrease around the 1990s, while Brazil and Cuba showed declines in ASRs in the last decade. Among females, total cancer mortality showed a favorable trend over the whole period in all the countries considered except Brazil, where the trend was stable.

**Fig. 3:**
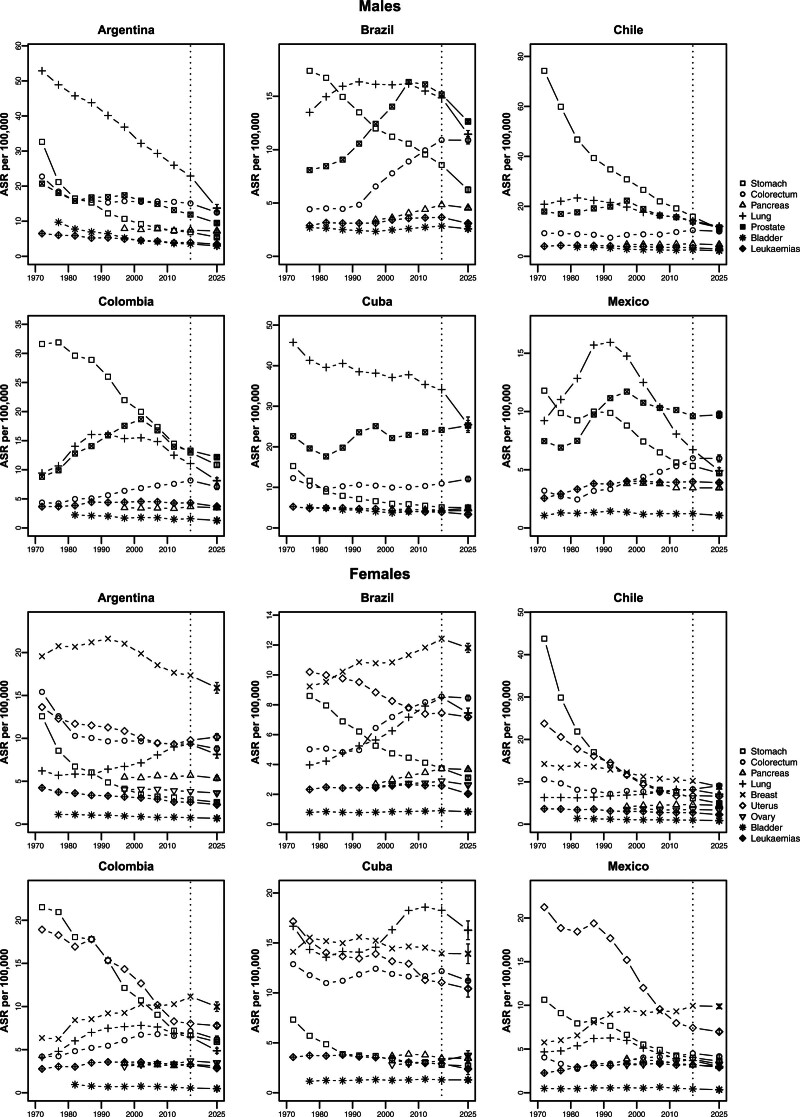
Age-standardized (world population) mortality rates (ASRs) per 100 000 males (top panels) and females (bottom panels) in quinquennia from 1970 to 2020 and predicted ASRs for 2025, with 95% prediction intervals for the considered cancer sites in the selected Latin American countries.

Figure 3 reports quinquennial ASRs mortality trends for each cancer site and country analyzed. Male stomach cancer ASRs have been declining appreciably since 1970 in all countries considered, although they remain comparatively high on a global scale. Prostate cancer mortality showed downward trends in all countries except Cuba over the last two decades. In all countries, colorectal cancer rates have either leveled off or started to decline in recent years. ASRs for lung cancer have been decreasing steadily in Argentina and Cuba. In Chile and Colombia, the decline began around the 1990s, while in Brazil and Mexico it started in the 1980s. Cuba continues to report the highest rates among the Latin American countries analyzed, with an expected ASR of 25.5/100 000 males. Bladder cancer and leukemias, which have the lowest ASRs, show stable trends over the period in all countries analyzed.

Female mortality from stomach and uterine cancer appreciably decreased in all Latin American countries considered throughout the period. Trends for colorectal cancer have been favorable in recent calendar years up to 2025, with the exception of Chile. Lung cancer ASRs among females increased between 2000 and 2010; trends have slowed in recent years, showing favorable predicted ASRs to 2025 in all selected countries.

Since 1970, breast cancer ASRs have been decreasing in Argentina, Cuba, and Chile, while Brazil, Colombia, and Mexico only show favorable trends over the most recent calendar years. Ovarian cancer, bladder cancer, and leukemias have been approximately stable over time.

Table 3 shows the ASRs for prostate cancer at all ages and in different age groups in 2010–2014 and 2015–2019 and the projected ASRs for 2025 in the six Latin American countries considered. Projected ASRs are around 4–5/100 000 males between 50 and 59 years of age, 30/100 000 between 60 and 69 years of age, and more than 100/100 000 between 70 and 79 years of age. The major exception is Cuba, where the expected ASRs are twice as high as in the other Latin American countries considered. In most countries, prostate cancer mortality has been declining with similar slopes in various age groups, except in Cuba and Mexico where there is no decline at age 80+.

**Table 3:** Age-standardized (world population) prostate cancer mortality rates (ASRs) per 100 000 males at all ages and at different age groups in selected Latin American countries during 2010–2014 and 2015–2019 periods and predicted ASRs for 2025 and the corresponding prediction intervals (PIs), along with percentage differences between 2015–2019 and 2025

	ASR 2010–2014	ASR 2015–2019	Predicted ASR 2025 (95% PI)	% Difference 2025 vs 2015–2019
Argentina				
All ages	13.18	11.90	9.50 (8.96–10.03)	−20.17
50–59 years	6.08	5.51	4.53 (3.73–5.32)	−17.79
60–69 years	40.82	37.59	30.99 (28.12–33.86)	−17.56
70–79 years	154.94	137.41	115.28 (106.41–124.15)	−16.11
80+ years	502.26	455.85	337.46 (296–378.93)	−25.97
Brazil				
All ages	16.12	15.19	12.64 (12.41–12.87)	−16.79
50–59 years	6.03	5.62	5.18 (4.76–5.60)	−7.83
60–69 years	38.86	37.98	31.72 (29.96–33.47)	−16.48
70–79 years	152.55	143.55	119.58 (115.23–123.93)	−16.70
80+ years	818.64	762.78	627.6 (614.07–641.14)	−17.72
Chile				
All ages	15.66	13.88	11.50 (10.90–12.10)	−17.14
50–59 years	5.53	4.86	4.35 (3.18–5.51)	−10.49
60–69 years	41.15	38.10	31.95 (28.73–35.18)	−16.14
70–79 years	178.59	162.92	120.86 (110.6–131.13)	−25.82
80+ years	687.11	583.71	519.56 (474.22–564.9)	−10.99
Colombia				
All ages	13.96	13.30	12.17 (11.71–12.63)	−8.50
50–59 years	4.97	4.48	4.58 (3.83–5.33)	2.23
60–69 years	34.28	32.47	27.64 (24.76–30.52)	−14.88
70–79 years	141.11	133.28	122.95 (116.06–129.85)	−7.75
80+ years	681.04	655.83	608.30 (573.36–643.25)	−7.25
Cuba				
All ages	23.59	24.17	25.24 (24.00–26.49)	4.43
50–59 years	12.15	11.45	11.11 (9.19–13.02)	−2.97
60–69 years	73.99	75.80	70.92 (63.70–78.14)	−6.44
70–79 years	272.52	281.73	290.48 (266.48–314.48)	3.11
80+ years	902.31	929.16	1038.35 (952.2–1124.51)	11.75
Mexico				
All ages	10.13	9.59	9.72 (9.40–10.03)	1.36
50–59 years	5.46	5.09	4.82 (4.29–5.34)	−5.30
60–69 years	32.87	31.43	29.86 (28.24–31.48)	−5.00
70–79 years	122.45	114.60	105.68 (99.93–111.44)	−7.78
80+ years	359.03	343.49	395.64 (372.4–418.88)	15.18

**Fig. 4:**
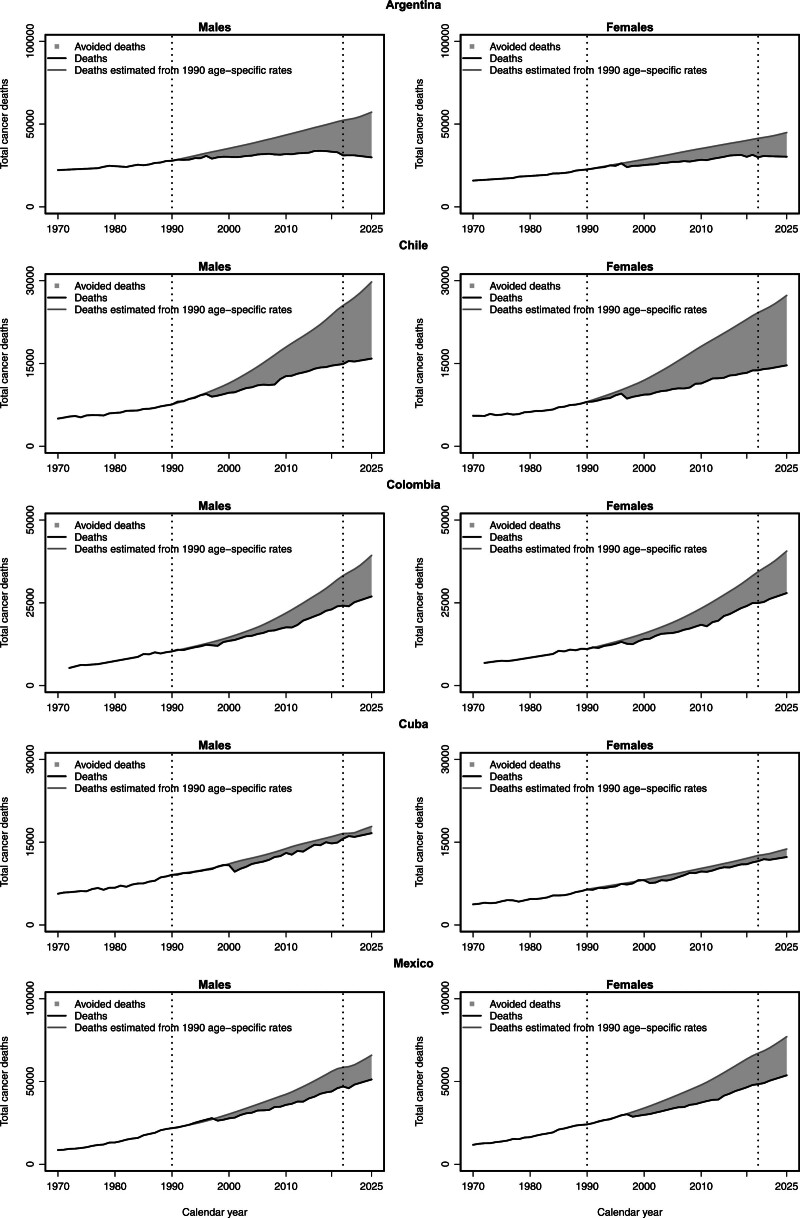
Total number of cancer deaths averted for five of the Latin American countries considered, in both sexes, between the peak rate in 1990 and 2025 (light gray area); observed and projected number of cancer deaths from 1991 to 2025 (black line); estimated number of total cancer deaths by applying the 1990 age-specific peak mortality rates (gray line). Over the 35-year period, a total of about 1 908 900 cancer deaths were averted in five of the six countries considered (957 100 males and 951 800 females). No reduction in cancer deaths was observed in Brazil. In 2025 alone, about 69 400 deaths among males and about 64 600 among females are projected to be avoided in the countries considered, none of them in Brazil.

Figure 4 shows the estimated number of cancer deaths averted in males and females between 1991 and 2025, assuming constant age-specific rates in 1990 (light gray area). Over the 35-year period considered, we estimated a total of more than 595 200 averted cancer deaths in Argentina (382 000 males and 213 200 females), 387 400 in Chile (184 200 males and 203 200 females), 320 800 in Colombia (150 900 males and 169 900 females), 49 900 in Cuba (27 400 males and 22 500 females), and 555 500 in Mexico (212 600 males and 342 900 females). No reduction in cancer deaths was estimated for Brazil.

## Discussion

The downward trend in mortality from all cancers combined is projected to continue in most Latin American countries through 2025 for both sexes. The highest projected rates were observed in Cuba and the lowest in Mexico for both sexes. There was also a tendency toward a levelling off of overall cancer mortality rates across countries, reflecting more uniform exposure to lifestyle and risk factors, with the main exception of Cuba.

The highest observed and predicted mortality rates for prostate cancer were in Cuba, exceeding 25/100 000 males. These rates have continued to rise over the last two decades, placing Cuba among the countries with the highest prostate cancer mortality worldwide. In the other countries analyzed, rates remained somewhat higher than those observed in Europe (Bertuccio *et al*., 2021), but showed decreasing trends since 2000. Worldwide, the highest incidence rates were registered in Australia, New Zealand, North America, Brazil, and several countries in northern Europe (Schafer *et al*., 2024), largely related to the widespread use of prostate-specific antigen testing. In contrast, the highest mortality rates were observed in Cuba, other Latin American and Caribbean countries, reflecting – besides genetic factors – limited access to early detection and treatment. In Latin America, particularly in Cuba, the high proportion of people of African descent, who have a greater genetic predisposition, is the main contributor to high cancer incidence and mortality rates (Reis *et al*., 2020; Conti *et al*., 2021; Angel *et al*., 2024). Chemoprevention with 5-alpha reductase inhibitor may have limited impact on prostate cancer rates (Gandaglia *et al*., 2021), but the key reason for the recent favorable trends in mortality are advancements in therapy over the last two decades, starting from Abiraterone and other androgen blocking therapies (Desai *et al*., 2021) to more recent immunotherapy (Wang *et al*., 2022). In addition, recent studies showed that the combination of chemotherapy or novel androgen receptor-targeting agents with androgen deprivation therapy can enhance oncologic outcomes (Zattoni *et al*., 2023). Access to these innovative treatments remains in most Latin America countries, particularly in Cuba.

With the exception of Cuban males, rates of stomach cancer are predicted to fall in 2025 compared to 2020 in all countries considered and for both sexes, following long-term favorable trends (Santucci *et al*., 2023). In particular, the observed and predicted rates in Cuba and Colombia, but also other Latin America countries, are higher than those observed in Europe (Collatuzzo *et al*., 2023) or North America (Siegel *et al*., 2024). The prevalence of *Helicobacter pylori (HP*) infection, the main risk factor for stomach cancer, has been declining globally and in the Americas since the 1980s (Chen *et al*., 2024), but in 2015 it was still around 50% in Argentina and Mexico and over 70% in Brazil and Chile in 2015 (Hooi *et al*., 2017). Eradication of *HP* at a young age plays a role in reducing the incidence of gastric cancer (Curado *et al*., 2019) and is therefore a possible prevention strategy in Latin America, together with treatment of symptomatic carriers (Ford *et al*., 2020).

Colorectal cancer mortality rates and trends in Latin American countries are in line with those in Europe (Santucci *et al*., 2024a). Over the last 5 years, rates are projected to increase in Brazil and Cuba for males and in Chile for females. As in Europe and the USA, the increased prevalence of overweight and obesity is likely to have an impact on colorectal cancer mortality particularly in the young not subject to screening and early diagnosis (Malik *et al*., 2020; Ferreira *et al*., 2024; Santucci *et al*., 2024b; Sung *et al.* 2025). On the other hand, long-term reduction depends on improved treatment and early detection through screening, which should be increased in Latin America (Montalvan-Sanchez *et al*., 2024).

Pancreatic cancer mortality rates in Latin America remain lower than in Europe and the USA and the trends are predicted to level off except for males from Argentina and Cuba where it increased slightly. Smoking prevalence, the main risk factor for pancreatic cancer, which has been relatively low in Latin American countries except Cuba, can explain only part of the observed trends (Blanco *et al*., 2014; Lugo *et al*., 2018). However, advances in early diagnosis, treatment, and management of the disease would have a significant impact on these mortality trends, but these remain limited (Hu and O’Reilly, 2024).

Despite the favorable trends in mortality, lung cancer remains the first cause of cancer-related deaths in Latin America as well as globally, though most Latin American countries (except Cuba) have comparatively low lung cancer mortality. This again highlights the importance of tobacco control in Cuba (Blanco *et al*., 2014).

The lower bladder cancer mortality in Latin America, compared with other geographical areas, can again be related to a lower frequency of smoking. Arsenic contamination of water in selected areas of Chile and Argentina may partly explain their relatively high bladder cancer mortality rates (Litter *et al*., 2019; Khan *et al*., 2020; Alam *et al*., 2023).

Mortality from breast cancer in Latin America is predicted to be favorable, reflecting improvements in the diagnosis and treatment of the disease, despite recent unfavorable changes in reproductive and anthropometric factors (overweight and obesity) in these countries (Romieu *et al*., 2018; His *et al*., 2020).

Ovarian cancer mortality is decreasing in all countries except Cuba. These patterns are largely attributed to the increased use of oral contraceptives in Latin American countries (Ugaz *et al*., 2015), which offer long-term protective effects against ovarian cancer (La Vecchia, 2017; Jara-Rosales *et al*., 2024). Recent advances in diagnosis and treatment may have played a minor role on mentioned mortality rates (Kuroki and Guntupalli, 2020; Musacchio *et al*., 2022), although access to innovative treatments in Latin America is limited (Giornelli *et al*., 2021).

Uterine cancer mortality shows favorable trends in all countries except Argentina. The high uterine cancer mortality reflects the high incidence of cervical cancer in most Latin American countries (Ferlay *et al*., 2024). In fact, cervical cancer mortality rates were higher in Latin America, with exceedingly high rates (above 10.0/100 000) in Argentina and Cuba, than in North America or Europe and cervical cancer screening [Pap smear and human papillomavirus (HPV) testing] has long been inadequate. The introduction of Pap test and HPV screening, as well as HPV vaccination across Latin America is therefore a priority.

Leukemias mortality projections remain favorable or stable, except for males in Mexico and females in Colombia and Chile. Rates remain high compared to most high-income countries, probably due to delays in the introduction of innovative treatments and inequalities in access to health care (Chiattone *et al*., 2020).

Our predicted estimates should be interpreted with caution due to the limitations of the models, which are not designed to capture recent changes in slopes. However, the analysis focuses on large and medium size countries, minimizing problems of excessive random variation. With observed cancer deaths for 2021 now available in the WHO database, we compared these rates with our previous predictions for 2023 and found that, for all cancers combined, the prediction errors were less than 5%, except for Argentina. Another limitation of the present study is the lack of reliable data for some large Latin American countries, such as Peru and Venezuela. In addition, socioeconomic and demographic disparities (e.g. urban/rural) within Latin American countries are likely to correspond to disparities in cancer mortality, but this cannot be accounted for by the available national data.

Besides the priority of tobacco control, particularly in Cuba, improvements in cervical cancer prevention and control through HPV screening and vaccination, and advancements in cancer diagnosis and treatment, including prostate cancer, remain essential across Latin America.

## Acknowledgements

The scientific support of the European Cancer Prevention (ECP) Organization is acknowledged.

This work was supported by the AIRC Foundation, Italy [grant no. 22987]. C.S. and C.L.V. were also supported by the EU funding within the NextGenerationEU-MUR PNRR Extended Partnership initiative [no. PE00000007, INF-ACT]. The funding sources had no role in the design and conduct of the study; collection, management, analysis, and interpretation of the data; preparation, review, or approval of the manuscript; and the decision to submit the manuscript for publication.

### Conflicts of interest

There are no conflicts of interest.

## Supplementary Material


