# Supplementary material for: Cancer mortality predictions for 2025 in Latin America with focus on prostate cancer
Source: Eur J Cancer Prev. 2025 Feb 25;35(2):97–107. doi: 10.1097/CEJ.0000000000000959 (PMC12851545; doi:10.1097/CEJ.0000000000000959)
Supplement: Supplementary file 1 [file ejcp-35-097-s001.pdf]

SUPPLEMENTARY TABLE

Table S1. List of malignant neoplasms and the corresponding diagnostic codes**.**

Neoplasm	ICD-10 code
Stomach	C16
Colorectum	C17-C21, C26
Pancreas	C25
Lung	C33-C34
Breast	C50
Uterus	C53-C55
Ovary	C56
Prostate	C61
Bladder	C67
Leukemias	C91-C95
All cancers	C00-D48

ICD: International Classification of Diseases.
